# The stroke care puzzle: Does tracheostomy timing fit?

**DOI:** 10.1186/s13054-023-04482-x

**Published:** 2023-06-01

**Authors:** Christopher Camarda, Lavienraj Premraj, Paolo Pelosi, Sung-Min Cho, Denise Battaglini

**Affiliations:** 1 Griffith University School of Medicine, Southport, QLD Australia; 2 Critical Care Research Group, The Prince Charles Hospital, Chermside, QLD Australia; 3 IRCCS Ospedale Policlinico San Martino, Genoa, Italy; 4 Department of Surgical Sciences and Integrated Diagnostics (DISC), University of Genoa, Genoa, Italy; 5 Division of Neurosciences Critical Care, Department of Neurology, Neurosurgery, Anaesthesiology and Critical Care Medicine and Neurosurgery, Johns Hopkins University School of Medicine, Baltimore, USA

We thank Drs. Sutt and Fraser for their insightful commentary on our publication. They open an important debate on tracheostomy timing and outcomes of interest.

We concur that for decades, research has focused on “traditional” outcomes such as mortality and length of stay (LOS). Additionally, focus on tracheostomy-specific outcomes including swallowing, and communication may aid efforts to improve recovery after intensive care unit (ICU) discharge.

Our analysis of > 17,000 critically ill patients with severe stroke attempted to clarify whether tracheostomy timing is an important piece of the stroke management puzzle [1]. Early tracheostomy (< 5 days from initiation of mechanical ventilation) was not associated with “traditional” outcomes or neurological outcome Modified Rankin Scale (mRS) [1]. Exploratory analysis showed that compared to Glasgow Coma Scale (GCS) on admission and stroke type, timing was not an important predictor of outcome. This was relatively unsurprising given all patients had moderate to severe stroke (recorded National Institutes of Health Stroke Scale (NIHSS) > 16) and overall GCS on admission of 7 [95% confidence interval = 5.95–7.67] [1]. Protracted LOS and poor prognosis are common in such patients, despite concerted efforts [2].

We found that early tracheostomy does not impact neurological outcome (mRS). This was reflected in the SETPOINT2 trial, where 75% of patients in each group experienced poor neurological outcome (mRS ≥ 4 at 6 months [early: 75%, late: 81%]) despite active rehabilitation efforts (~ 70% of patients were discharged to rehabilitation facilities) [2].

We recognise that, strictly speaking, the timing of tracheostomy should not substantially impact neurological outcome. Studies often inadequately separate early and late groups (SETPOINT2 < 5 days, > 10 days [2]; TracMAN ≤ 4 days, > 10 days [3]). The physiological link between neurological recovery and simple surgical procedure performed a few days earlier is tenuous. In this context, all-encompassing outcomes such as mRS (capturing independent mobility, incontinence, and attending to bodily needs) [4] help define how patients have survived, thrived, and preserved their dignity throughout their recovery. As such, patient perspectives were not entirely lost in our analysis [1].

Evidence on patient-centric outcomes in this population is limited. Our work humbly sought to quell decades of debate on the impact of tracheostomy timing on mortality, neurological outcome (mRS), and LOS, in patients with severe stroke. Given our findings, we commend future efforts to protocolise reporting of additional patient-centred outcomes such as speech (Time to Vocalisation), swallowing (Functional Dysphagia Scale) [5] and psychological outcomes (Hospital Anxiety and Depression Scale) in future randomised controlled trials, to better inform patient-centred clinical decision-making.

## Data Availability

Not applicable.
